# A Combined Extract Derived from Black Sticky Rice and Dill Improves Clinical Symptoms and Ischemic Stroke Biomarkers in Transient Ischemic Attack and Ischemic Stroke Patients

**DOI:** 10.3390/nu16223946

**Published:** 2024-11-19

**Authors:** Kannikar Kongbunkiat, Wipawee Thukham-mee, Somsak Tiamkao, Narongrit Kasemsap, Nisa Vorasoot, Jintanaporn Wattanathorn

**Affiliations:** 1 Department of Medicine, Faculty of Medicine, Khon Kaen University, Khon Kaen 40002, Thailand; kannikarkon@kku.ac.th (K.K.); somtia@kku.ac.th (S.T.); naroka@kku.ac.th (N.K.); nisa@kku.ac.th (N.V.); 2 North-Eastern Stroke Research Group, Khon Kaen University, Khon Kaen 40002, Thailand; 3 Research Institute for High Human Performance and Health Promotion, Khon Kaen University, Khon Kaen 40002, Thailand; meewep@gmail.com; 4 Department of Physiology, Faculty of Medicine, Khon Kaen University, Khon Kaen 40002, Thailand

**Keywords:** ischemic stroke, the combined extract of black sticky rice and dill, stroke biomarkers

## Abstract

Currently, the adjuvant therapy to optimize the restorative process after stroke is required due to the unsatisfied therapeutic efficacy. A combined extract of black sticky rice and dill showed potential in the preclinical state, so we hypothesized that it could provide clinical benefits. A three-arm, randomized, placebo-controlled study was set up to elucidate this issue. Both males and females (18–80 years old) who had experienced transient ischemic attacks or ischemic strokes within the last 5–10 days with an NIHSS score ≤ 7 and received standard treatment were randomly assigned to receive either a placebo or capsule containing a combined extract of black sticky rice and dill at a dose of 600 or 1200 mg per day. The safety parameters, movement control, and degree of disability were assessed 1, 2, and 6 weeks after the intervention, and serum stroke biomarkers were assessed at the mentioned time points, except at 2 weeks. After week 1, the high-dose (1200 mg/day) treatment group had improved NIHSSS, VCAM1, and MMP-9. Both S100β and VCAM1 also improved at week 6, while the low-dose treatment group (600 mg/day) only exhibited improved VCAM1. Therefore, a high dose of the developed adjuvant supplement improves stroke recovery by improving motor impairment by reducing endothelial dysfunction and inflammation.

## 1. Introduction

Acute ischemic stroke constitutes a significant global health challenge, accounting for approximately 85% of all stroke cases and representing a leading cause of mortality and long-term disability worldwide. With an estimated global burden of 16.9 million new stroke cases each year, the condition not only precipitates a high mortality rate but also leaves a majority of survivors with substantial disabilities, necessitating immediate and efficacious emergency treatment to improve clinical outcomes [1]. A recent study reported that global ischemic stroke cases reached around 9.62 million in 2020 [2]. Therefore, this issue is continually rising in importance. Current therapies, such as thrombolysis and thrombectomy to provide rapid revascularization, have been revealed to markedly reduce the severity of disability. However, the therapeutic efficacy is still unsatisfactory due to the short golden period of treatment [3,4], emphasizing the critical nature of timely intervention [5]. Most stroke patients cannot be managed in the golden period. Thus, around 45.2–60% of stroke survivors experience disability and poor quality of life [6,7]. Owing to the aforementioned therapeutic gap, adjuvant therapy with herbal medicine has gained significant attention [8,9] based on the belief that herbs, a natural product, produce effective outcomes with less toxicity. This is supported by a recent systematic review demonstrating that polyphenol- and/or flavonoid-enriched herbal substances can be applied safely due to low toxicity and high efficacy. In addition, they are a potentially indispensable strategy [9].

The purpose of the combined treatments between the standard treatment pipeline and adjuvant therapy is to improve the outcome by promoting repair and restorative processes [10]. To optimize the efficiency of the restorative process, an understanding of the cellular level is necessary. However, an assessment of the cellular events underlying the recovery process in humans is inaccessible. Therefore, serum biomarkers, which are easier to access and provide deeper insight into spontaneous recovery in humans, are used as surrogate markers in clinical trials of restorative therapies. Current stroke recovery biomarkers reflect the pathophysiological changes that occur during a stroke, such as inflammation, neuronal injury, oxidative stress, astrocyte activation, endothelial dysfunction, and hemostasis [11]. Currently, these markers not only serve as surrogate markers reflecting the events related to stroke pathophysiology and restorative process but also serve as therapeutic targets [12].

A significant amount of evidence has demonstrated that the main mechanisms of ischemic pathophysiology and the restorative process are associated with oxidative stress [13,14] and inflammation [14,15]. The brain blood supply interruption induces oxygen and glucose deprivation (OGD), which, in turn, disturbs mitochondrial functions, giving rise to an increase in oxidative stress. This change can stimulate autophagy, apoptosis, and glutamate excitotoxicity, resulting in neurodegeneration [16]. In addition, tissue injury induced by the disruption of blood supply activates microglia and infiltrating circulatory cells, such as granulocytes, neutrophils, macrophages, and T cells, leading to the release of proinflammatory chemokines and cytokines and giving rise to neurodegeneration. Furthermore, neurodegeneration induced by an inflammatory reaction can also be intensified by the elevation of matrix metalloprotease (MMP) stimulated by oxidative stress [17]. Among the mechanisms mentioned earlier, oxidative stress and inflammation are regarded as factors that play important roles in the restorative process following a stroke attack [14,18]. Owing to this information, the facilitating effect of substances possessing antioxidant and anti-inflammatory characteristics on neurorestorative processes has gained much attention. This is confirmed by findings that reveal the improved outcome of stroke recovery via improved plasticity due to food components with antioxidant and anti-inflammatory effects [14,19].

Black sticky rice (*Oryza sativa* L.) and dill (*Anethum graveolens*), which are local products in the northeastern region of Thailand, are reputed to have antioxidant and anti-inflammatory effects [20,21]. Due to the antioxidant and anti-inflammatory effects of the aforementioned substances, as well as their synergistic effect, we developed a combined extract of black sticky rice and dill to optimize the biological activity benefits mentioned above. Our results showed that the developed combined extract contains high contents of polyphenolic compounds (1724.10 ± 159.73 μg of GAE/mg extract) and revealed a synergistic interaction between both extracts, which was manifested by a combination index (CI) less than 1 (data are shown in the Supplementary Materials). Our preclinical study on an animal model of ischemic stroke with metabolic syndrome (MetS) revealed a neuroprotective effect against ischemic brain damage and dysfunction without toxic effects [22,23]. The underlying mechanisms are associated with the antioxidant and anti-inflammatory action of the main phytochemical ingredients, such as chlorogenic acid, ellagic acid, rutin, and anthocyanins (cyanidin-3-glucoside, or C3G, cyanidin-3-rutinoside, or C3R, and cyanidin-3-O-sophoroside, or C3S) [24,25,26,27,28], as shown in Table 1. The combined extract decreases inflammatory cytokines, such as interleukin-6 (IL-6) and nuclear factor-kappa B (NF-kB), together with a reduction in malondialdehyde (MDA), reflecting an improvement in inflammation and oxidative stress status, which play pivotal roles in the pathophysiology and recovery process of ischemic stroke. In addition, the median lethal dose (LD50) of the combined extract of black sticky rice and dill is more than 5 g/kg BW (unpublished data), which indicates that it is safe [29]. Owing to its safety and the potential benefit in the animal model mentioned earlier, we hypothesized that the application of a combined extract of black sticky rice and dill as adjuvant therapy could improve pathophysiology-associated events at the cellular level of ischemic stroke and transient ischemic attack (TIA) and lead to an improvement in clinical manifestations. However, confidence in its benefits for patients with acute ischemic stroke and transient ischemic attacks (TIA) cannot be assured due to potential species and lifestyle differences, as well as variations in consumed medications. To test our hypothesis, we aimed to determine the effect of adjuvant therapy with the supplement containing the combined extract of black sticky rice and dill on the clinical manifestation outcomes and pathophysiology-related serum biomarkers of ischemic stroke in patients with TIA and patients with acute ischemic stroke.

## 2. Materials and Methods

### 2.1. Preparation of the Health Product Containing the Combined Extract of Black Sticky Rice (Oryza sativa L. Var. Kum Yai) and Dill (Anethum graveolens)

Dill from Khon Kaen Province, Thailand, was harvested in February 2020 at 45 days old. It was authenticated by Dr. Pornchai Klutwong (a Taxonomist at the Faculty of Science, Khon Kaen University), and a voucher specimen (voucher specimen 25551) was kept at the Khon Kaen University Herbarium, Thailand. After washing, it was dried, ground to powder, and extracted with 95% ethanol using the maceration technique. The extract was then concentrated using an evaporator (Rotavapor^®^ R-100) and spray drier (Büchi B290 Mini Spray-Dryer), BUCHI Ltd., Bangkok, Thailand. The yielding percentage was 14%.

Black sticky rice was obtained from Amphoe Srithat, Udonthani Province, Thailand. After authentication by Dr. Pornchai Klutwong, herbarium specimen No. 25552 was also kept at the Khon Kaen University Herbarium, Thailand. The black sticky rice was heated at 50 °C for 1 h to reduce moisture, and it was prepared as a water extract using the maceration technique. Then, the extract was filtered and mixed with 1% maltodextrin before concentrating with a spray drier. The yielding percentage was 22%.

To develop the combined extract, both extracts were mixed at an appropriate ratio (a trade secret) to provide a synergistic interaction. The concentrations of the main phenolic compounds in the combined extract are shown in Table 1. Both the combined extract and placebo were prepared as capsules by DOD Biotech Public Company Limited, Mueang Samut Sakhon District, Samut Sakhon 74000, Thailand. The compositions of the placebo and capsule containing the combined extract of black sticky rice and dill are shown in Table 2.

The quality was then assessed against the US Pharmacopeia Standard USP 36 criteria. The developed product met the standard criteria.

### 2.2. Study Design

This study was a 3-arm, randomized, double-blind, placebo-controlled study. All procedures in this study were approved by the Center for Ethics in Human Research, Khon Kaen University, Khon Kaen Province, Thailand (HE631634). The protocol was also registered with the Thai Clinical Trials Registry (TCTR20201224001). This study was performed in accordance with the International Conference of Harmonization (ICH) for Good Clinical Practice (GCP) and in compliance with the Declaration of Helsinki and its further amendments. Before participating in this study, all subjects provided a written consent form after attending the explanation section about the study’s purpose, all processes of this study, the participant’s duties, and the rights of the participant by the physician of the Neurology Division, the Department of Internal Medicine, the Faculty of Medicine, Khon Kaen University.

#### Sample Size

This study is a proof-of-concept study that was designed to determine the therapeutic effect of applying a combined extract of black sticky rice and dill as adjuvant therapy in patients with acute ischemic stroke with minor severity and transient ischemic attack (TIA). Therefore, the primary outcome should be related to the markers that are related to the pathophysiological changes of ischemic stroke and provide the most useful information, so we selected metalloproteinase-9 (MMP-9) because it is a validated biomarker that is associated with neurological deficit, middle cerebral artery occlusion, and infarct volume [30]. Therefore, the reference used for calculating the sample size was the article related to serum MMP9 [31]. The study design used repetitive measurement of the outcome parameters, so we used the sample size calculation formula shown in the following equation:

N/group = (Z_1−α/2_ + Z_1−β_)^2^ σ^2^ (r + 1)[1 + (T − 1)ρ/V^2^rT

In this study, we assume that *p* < 0.05 is significant or that we accept that the probability of difference in studying targets due to chance or false positive results is 5% (type 1 error or alpha). Therefore, Z_α/2_ is 1.96 for a type I error at 5%.

The type II error, or power, or β, according to the bio-statistical literature, is 0.20 or a 20% chance that the null hypothesis is falsely accepted. The “power” of this study is equal to (1 − β), and for a β of 0.2, the power is 0.8, which is the minimum power required to accept the null hypothesis.

σ is the value of the standard deviation of the experimental group. According to a study regarding the MMP-9 level after treatment with 500 mg of bromelain [31], it was 0.94.

r is the value of the ratio between the control and experimental groups. In this study, the value of r was 1.

T is the number of follow-up visitations, and the value of T in this study was 3.

ρ is a repeated measures correlation; in this study, it was set to 0.9 based on a report about the association between data of MMP-9 and various parameters, such as neurological impairment brain infarction, as mentioned earlier [30].

V is a value of the difference between the means of the control and experimental group that is meaningful in the clinic. In this study, the value was 1.

N/arm = [(1.96 + 0.8)^2^0.94^2^](1 + 1)[1 + (3 − 1)0.9] = 13.16/arm

Therefore, we used 13 subjects/arm in this study.

### 2.3. Subjects

The recruited volunteers were selected through the solicitation of voluntary participation from patients with transient ischemic attack and ischemic stroke who were treated at Srinagarind Hospital (a tertiary care and university hospital). Eligible subjects were male and female between 18 and 80 years old. They experienced transient ischemic attacks or ischemic strokes within the last 5–10 days, resulting in unilateral body weakness, with a National Institutes of Health Stroke Scale or NIHSS score of ≤7, but they were still able to communicate. All participants signed a written consent form after the details of this study had been fully explained to them by the study team. Exclusion criteria included a patient experiencing thrombolytic therapy (rtPA) or carotid endarterectomy (CEA), an NIHSS score of >7, a depression score, according to the Montgomery Åsberg Depression Rating Scale (MADRS), of >19, and the receipt of monoamine oxidase inhibitors, neuroleptic drugs, or benzodiazepines within one month prior to screening. In addition, pregnant women, diabetic patients with warfarin treatment, and those with chronic illnesses that make screening and monitoring challenging were also excluded. All subjects were randomly assigned to one of the following groups: (1) placebo group, or a group that receives the primary treatment medication and a placebo; (2) low-dose group, or a group that receives the primary treatment medication and a capsule containing the combined extract of black sticky rice and dill (600 mg/day); or (3) high-dose group, or a group that receives the primary treatment medication and a capsule containing the combined extract of black sticky rice and dill (1200 mg/day). All subjects underwent an assessment of safety parameters, including changes in hematologic parameters, clinical chemistry parameters, serum biomarkers regarding ischemic pathophysiology of ischemic stroke, and anthropometry changes at baseline and after 1 and 6 weeks of intervention, whereas brain clinical symptoms, such as NIHSS, were assessed at baseline and after the intervention at 1, 2, and 6 weeks.

### 2.4. Blood Sampling Collection and Plasma Preparation

Venous blood was collected in a plastic tube containing ethylenediaminetetraacetic acid (EDTA), which served as an anticoagulant. Samples were kept in an ice box and then subjected to 3000× *g* centrifugation at 4 °C for 20 min. At the end of the centrifugation process, an aliquot of plasma was harvested and stored at −20 °C until the time of analysis (within 2 months).

### 2.5. Safety Parameter Assessment

Blood samples were collected from the cubital vein, either on the right or left forearm, amounting to 12 milliliters each, to conduct a complete blood count (CBC), including hematocrit, hemoglobin concentration, erythrocyte count, reticulocyte count, total and differential leukocyte count, platelet count, and blood clotting time. Additionally, blood chemistry was also assessed for glucose, total cholesterol, HDL, LDL, triglycerides, blood urea nitrogen (BUN), creatinine, total protein, albumin, and electrolytes (sodium, potassium, bicarbonate, chloride). Organ function was also assessed via the determination of various enzyme activities, including alanine aminotransferase (ALT), aspartate aminotransferase (AST), alkaline phosphatase, gamma-glutamyl transpeptidase (Gamma-GT), and sorbitol dehydrogenase. Furthermore, the function of the endocrine system was also monitored via the assessment of T4, T3, TSH, FSH, and LH and the assessment of testosterone in males and estradiol in females. All parameters mentioned above were assessed at the Laboratory Unit Service, Srinagarind Hospital, the Faculty of Medicine, Khon Kaen University.

### 2.6. Measurement of Stroke Biomarkers

The goal of restorative therapy was to promote the repair process following a stroke attack [32]. Many serum biomarkers, including those associated with vascular injury, metabolic changes, brain injury, oxidative stress, and inflammation, are implemented to inform progress outcomes following treatment [33]. In this study, S100β was selected as a representative of biomarkers reflecting brain injury condition [34], whereas VCAM1 and vWF served as biomarkers reflecting endothelial dysfunction or changes in the blood vessel that supplies the brain [35,36]. Furthermore, MMP-9 was selected as the representative marker of inflammation [34,35]. The evaluation of stroke biomarkers included vascular cell adhesion molecule1 (VCAM1), Abcam Limited, Cambridge, UK, matrix metalloproteinases9 (MMP-9), Abcam Limited, Cambridge, UK, Von Willebrand factor (vWF), Abcam Limited, Cambridge, UK, and S100β (EZHS100B-33k), Merck, Darmstadt, Germany, in plasma (the preparation of plasma was mentioned in Section 2.3), which were measured using an ELISA ASSAY according to the protocols provided by the production company.

### 2.7. Assessment of Movement Control

The main outcome used to determine the intervention efficiency in this clinical trial treatment was neurological function and deficits, which were assessed using the National Institute of Health Stroke Scale (NIHSS) and corresponded with the previous study [37]. The degree of disability in stroke patients was also evaluated using a Modified Rankin Scale (mRS). The severity was graded between 0 and 6, where a low score, such as 0, indicated no symptoms and 6 indicated death [38].

Basic daily activities, including feeding, bathing, grooming, dressing, bowel control, bladder control, toileting, chair transfer, ambulation, and stair climbing, were assessed using the Barthel Index (BI). Each item was rated according to the level of care required and the ability to perform basic daily activities independently. The BI is a summation of all items. The index yields a total score of 100. The higher the score, the greater the degree of functional independence [39].

### 2.8. Statistical Analysis

Data are presented as mean ± standard deviation (SD). An analysis was conducted on the intention of treatment. Baseline data across three volunteer groups were compared by presenting numbers and percentages for categorical data and mean values, along with standard deviations for quantitative data. Changes in various indicators due to the product among the three groups of volunteers were compared using an analysis of variance (ANOVA) and Tukey’s post hoc tests. Comparisons of indicators after product consumption among the three groups of volunteers and adjusting for baseline values of these indicators were performed using ANCOVA and Tukey’s post hoc tests. Changes that were considered statistically significant were those with a *p*-value < 0.05.

## 3. Results

### 3.1. Participant Demographics and Characteristics

**Figure 1:**
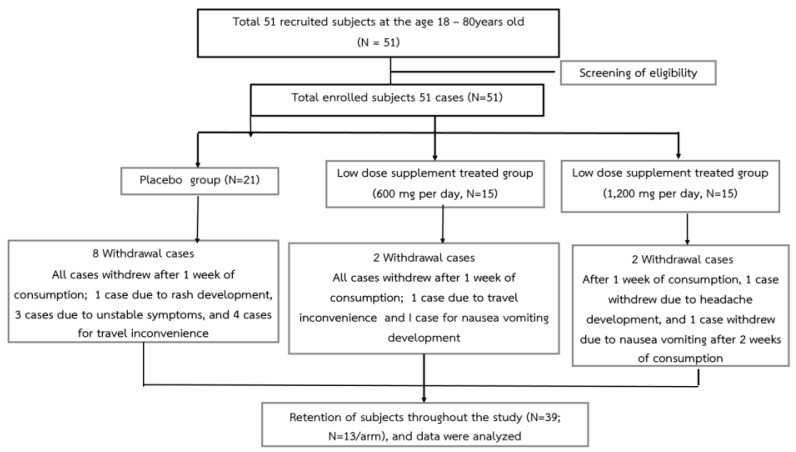
Flowchart of the subjects throughout this study.

The total recruited and eligible patients who fit the inclusion criteria totaled 51, with an average age of around 64.36 ± 2.40 years. They were randomly allocated to the placebo or two experimental groups that received a combined extract of black sticky rice and dill (600 and 1200 mg per day). Prior to the intervention, there were no significant differences in demographic data, including vital signs (temperature, blood pressure, and respiratory rate) and body mass index (BMI), detected among groups. A total of 13 retention cases per group completely participated in all processes until the end of this study. A total of eight subjects in the placebo group withdrew from this study after 1 week of treatment: one due to rash development and two due to unstable symptoms, which were defined as a worsening or fluctuation of stroke-related symptoms that required additional medical intervention or indicated a deterioration in clinical status. These included exacerbations of neurological deficits, such as increased weakness, speech difficulties, or cognitive impairment, that necessitated hospital admission or changes in standard care. These criteria ensured that participants who could no longer safely continue in the trial were appropriately withdrawn for their safety. Furthermore, four people withdrew due to travel inconvenience, which involved logistical issues that prevented participants from attending follow-up appointments. This could include factors such as transportation difficulties, long distances from the research center, or personal limitations that interfered with travel. These non-medical reasons for withdrawal were documented to highlight that they were not related to the safety or efficacy of the treatment. Regarding the low-dose treatment (600 mg per day) group, 2 of the 15 enrolled subjects withdrew from this study after 1 week of consumption, 1 withdrew due to travel inconvenience, and 2 withdrew due to the development of nausea and vomiting. The high-dose treatment (1200 mg per day) group also showed two withdrawal cases, one due to headache and dizziness development and one due to nausea and vomiting. The total number of retained cases in each group was 13, as shown in Figure 1. The distributions of male and female cases in the placebo, low-dose, and high-dose treatment groups were 4/9, 9/4, and 5/8, respectively, as shown in Table 3 and Table 4.

No significant statistical differences were found in body temperature, heart rate, respiratory rate, blood pressure, and body mass index (BMI), as shown in Table 3. The current data revealed that during the 1-week consumption period, no significant changes in all parameters in basic information mentioned earlier were observed. After 2 weeks of consumption, subjects who consumed the high dose of the combined extract of black sticky rice and dill had a significantly increased respiration rate (*p*-value < 0.05) compared to the placebo group. However, this change was still in the normal range. When the consumption period was extended to 6 weeks, no significant change in this parameter and other parameters regarding the basic information were observed, as shown in Table 4.

### 3.2. Safety Evaluation

To ensure consumption safety, we also determined all safety parameters, including hematologic and clinical chemistry parameters. Table 5 revealed that prior to the intervention, no significant differences in any parameters regarding hematologic changes were observed. After 1 week of consumption, subjects who consumed the combined extract of black sticky rice and dill at a dose of 600 mg/day had significantly increased red blood cells but decreased monocytes (*p*-value < 0.05 all). These significant changes disappeared at 6 weeks of consumption. However, all observed changes are still in the normal range.

Prior to the intervention, all parameters regarding clinical chemistry values and the atherogenic index in plasma (AIP), which indicated cardiovascular risk, failed to show significant differences, as shown in Table 6. After 1 week of consumption, subjects who consumed the combined extract of black sticky rice and dill at a dose of 600 mg/day had slightly increased LDL-C (*p*-value < 0.05, compared to the placebo group), whereas subjects who consumed the developed product at a dose of 1200 mg/day had a significantly increased creatinine level (*p*-value < 0.05) compared to the placebo group. When the consumption period was extended to 6 weeks, subjects who consumed a low dose of the combined extract of black sticky rice and dill had a significantly decreased LH level (*p*-value < 0.05) compared to the placebo group. All mentioned changes observed in this study were still in normal ranges, as shown in Table 7.

### 3.3. Effect of the Combined Extract of Black Sticky Rice and Dill on Clinical Symptoms

We assessed the effect of the developed product on the control of patient movement using the National Institute of Health Stroke Scale (NIHSS). Owing to the significant difference in the ability of patients to control movement at baseline, we reported data in this part as percentage changes from baseline. Table 8 clearly reveals that subjects who consumed the combined extract at a dose of 1200 mg/day experienced a significant improvement in movement control after 1 week of consumption (*p*-value < 0.05) compared to the placebo group. When the intervention period was prolonged, no significant changes were observed, and most patients recovered to normal conditions.

The effect of the combined extract of black sticky rice and dill on the ability to perform basic activities of daily living (ADLs) was also assessed using the Barthel Index (BI), and data are shown in Table 9. The current data demonstrate that prior to commencing the intervention, most basic daily activities, including feeding, bathing, grooming, bowel control, bladder control, toileting, chair transfer, ambulation, and stair climbing, showed no significant difference. The BI of subjects who consumed the combined extract of black sticky rice and dill at a dose of 1200 mg/day was higher than that of the placebo group (*p*-value < 0.05). After the commencement of the intervention, no significant change in BI among groups was observed throughout the 6-week study period.

The degree of disability in stroke patients was also evaluated, and data are indicated in Table 9. Prior to the intervention, no significant difference in stroke severity was observed among the groups. Subjects who consumed the combined extract of black sticky rice and dill at a dose of 1200 mg/day experienced a significantly improved degree of disability (*p*-value < 0.05) compared to placebo control after 1 week of consumption, as shown in Table 10.

### 3.4. Effect of the Combined Extract of Black Sticky Rice and Dill on Ischemic Stroke Biomarkers

Table 11 revealed that prior to the intervention, there were no significant differences in VCAM-1, MMP-9, vWF, and S100β. After 1 week of consumption, subjects who consumed the combined extract at a dose of 1200 mg/day experienced significantly decreased VCAM-1 and MMP-9 (*p*-value < 0.05, compared to the placebo group). Subjects who consumed the combined extract of black sticky rice and dill at a dose of 600 mg/day experienced a significantly decreased VCAM-1 (*p*-value < 0.05) compared to the placebo group, whereas subjects who consumed the combined extract of black sticky rice at a dose of 1200 mg/day experienced a significant reduction in both VCAM-1 and S100β (*p*-value < 0.05) compared to the placebo group.

## 4. Discussion

This is the first clinical study that clearly demonstrates the beneficial outcomes of a combined extract of black sticky rice and dill. This novel herb-based product improves motor control evaluated using the NIHSS and stroke serum biomarkers, such as MMP-9, VCAM-1, and s100β, in patients who experienced transient ischemic stroke and minor ischemic stroke.

The current study demonstrates that subjects who received a high dose of the combined extract of black sticky rice at a dose of 1200 mg showed an improvement in the NIHSS, the primary endpoint used for decision in this study, after 2 weeks of intervention. Then, no significant change in this parameter was observed. A possible explanation for this phenomenon may be associated with the changes that occur in various phases of the recovery process after a stroke attack. In the repair process, neuroplasticity is essential. It has been reported that the peak of changes involving neuroplasticity is in the early subacute phase post-stroke [38,39]. During the first week after a stroke attack, in the early subacute phase, numerous changes associated with plasticity occur. Then, all changes involving neuroplasticity decline; therefore, after 1 week of a stroke attack, we cannot detect a significant change in NIHSS improvement. This change corresponds with our previous clinical data that show an improvement in neurological deficit and cognitive function. Regarding the Barthel Index, a significant change in dressing ability was detected prior to the intervention. After the treatment, no significant change in this parameter was observed. However, when considering the improvement, the change fails to show a significant difference. The possible explanation for this discrepancy between the NIHSS and Barthel Index may be due to the Barthel Index being less sensitive and that the main focus of the Barthel Index is the ability to perform daily activities, whereas the NIHSS is sensitive to all aspects of stroke severity, including consciousness, facial expression, limb movement, language, sensory experience, and visual field. In this study, only the high dose exhibited a positive modulation effect. The low dose failed to show a significant improvement in the NIHSS, which reflects an improvement in stroke severity. This may be associated with the low concentration of the possible active ingredient, which may fail to achieve the therapeutic level. Owing to the improvement in the primary endpoint or NIHSS, we suggest that the combined extract of black sticky rice and dill shows the potential to serve as adjuvant therapy.

It has been reported that the improvement in stroke severity is associated with neuroplasticity [40], which, in turn, relates to VCAM-1 [41,42], MMP-9 [43], S100β [44], and vWF [45]. Therefore, we also assessed the alterations of these biomarkers. Data obtained from animal model studies revealed that within the first 24 h after a stroke, the serum proMMP-p level increases. This change is associated with the volume of brain ischemia [46]. Additionally, during the first 48 h, an increase in MMP-9 not only relates to the volume of brain ischemia but also correlates with the observed neurological deficits [47]. Moreover, high levels of MMP-9 have been found between 7 and 14 days post-stroke [48]. Therefore, the finding that volunteers who consumed a 1200 mg capsule of black sticky rice and dill extract for one week showed a significant improvement in the severity of NIHSS assessed using the NIHSS and post-stroke disability levels (mRS) could be partly due to a reduction in MMP-9 levels. This is in line with studies that reported a decrease in MMP-9 after patients with acute ischemic stroke received 500 mg of bromelain daily for 14 days, which correlated with clinical improvement [31]. MMP-9 plays a critical role in reducing apoptosis and brain tissue destruction [49,50,51,52], in addition to causing proteolysis and blood–brain barrier disruption, leading to brain edema and influencing inflammation through leukocyte infiltration [31]. However, by 6 weeks, all groups showed improvement, so no significant differences were observed. Furthermore, MMP-9, which involves neuroplasticity [53], has a peak in the early subacute phase post-stroke [38,39]. During the first week after the stroke attack, in the early subacute phase, numerous changes associated with plasticity occur. Then, all changes involving neuroplasticity declined, so after 1 week of the stroke attack, we could not detect a significant change in NIHSS improvement. Currently, MMP-9 is considered a new target in ischemic stroke treatment [48].

Beyond the changes in MMP-9 mentioned above, ischemic stroke patients have been found to exhibit increased levels of soluble (s) intercellular and vascular cellular adhesion molecules-1, particularly VCAM-1 [54]. The serum levels of VCAM-1 reflect changes in the brain and correlate with brain infarct volume and brain edema volume, which affect the reduction of stroke severity [55]. Thus, the decrease in VCAM-1 observed in this study may be related to the volunteers who consumed the black sticky rice and dill extract capsules at 1200 mg for 6 weeks. However, this should not be considered the main factor since volunteers consuming a 600 mg dose also showed a reduction in VCAM-1 but without a significant improvement in the NIHSS and disability levels (mRS). This is consistent with the report by Justicia et al. (2006) [56], who found that VCAM-1 antibodies could not prevent brain damage in ischemic stroke patients.

Regarding serum S100β, a protein marker reported to be associated with the volume of brain ischemia, stroke severity, and functional outcome and indicative of changes in brain volume and clinical outcome [57], the improvement in disability conditions after consuming the black sticky rice and dill extract capsules at 1200 mg for 6 weeks may be partly due to a reduction in the volume of brain ischemia and decreased serum S100β levels.

This study is the first clinical trial that demonstrates the beneficial effect of a combined extract of black sticky rice and dill as adjuvant therapy for ischemic stroke. This novel herb-based product improves motor control as evaluated using the National Institutes of Health Stroke Score (NIHSS); level of post-stroke disability; and stroke serum biomarkers, such as MMP-9, VCAM-1, and s100β, in patients who experience transient ischemic and minor ischemic stroke. Owing to the previous findings on the roles of MMP-9 and VCAM1 in brain tissue damage, we suggest that the improvement in motor control observed in this study may partly associated with the reduction in MMP-9 and VCAM-1.

Previous research has indicated that chlorogenic acid, a major component of the tested product, protects against brain ischemia [58]. Therefore, the improvement in the disability conditions of the volunteers could be partly related to chlorogenic acid. In addition to chlorogenic acid, ellagic acid [59], rutin [60], and anthocyanin [61] also have a protective effect against cerebral ischemia. The substances mentioned above may also play contributing roles. Furthermore, the influence of interactions between different substances in the product cannot be excluded. A better understanding of the main active ingredients playing crucial roles is still required in further exploration.

Overall, the current study demonstrates that no unstable stroke symptoms were found in subjects who received the combined extract of black sticky rice and dill at low and high doses during adjuvant therapy throughout this study. Furthermore, no changes in hematologic parameters and clinical chemistry values reflecting toxicity signs were observed. Therefore, the developed product is less likely to produce serious toxic effects. However, some subjects developed nausea and vomiting in both experimental groups, at around 7.69% in each group. In addition, headache development and dizziness were also reported (7.69%) in the high-dose treatment group. These data suggest that the application of the combined extract as adjuvant therapy during an acute attack of ischemic stroke and TIA can produce non-serious side effects, such as nausea, vomiting, and headache, in some cases and should be used with precaution. Although this study shows no serious toxicity in humans, and the LD of the combined extract in this study is more than 5 g/kg BW, reflecting that this combined extract is practically safe, the interaction effect of this combined extract and the main current main pipeline drugs still requires further investigation. This study also has some limitations, such as a small number of subjects, so we cannot perform a subgroup analysis to provide a better understanding of the modulation effects of gender and lifestyle differences on epigenetic modification, which, in turn, induces different responses, such as nausea, vomiting, and headache, observed in this study. Furthermore, the budget constraints of this study do not allow us to determine these effects in depth. Therefore, this point requires further exploration.

## 5. Conclusions

This study examined the impact of black sticky rice and dill extract on ischemic stroke and transient ischemic attack patients, focusing on its potential therapeutic benefits as an adjuvant for principal treatments. The current results show that the combined extract of black sticky rice and dill significantly improved motor control and lowered specific biomarkers associated with stroke without any observed toxicity. The improvement in motor control observed in this study may be attributed to the reduction in MMP-9 and VCAM-1, which gives rise to an improvement in brain damage and a reduction in serum s100β, finally leading to an improvement in motor control. These results suggest the promising role of this combined extract as a functional food in adjunct therapy for stroke treatment and, potentially, for the prevention of vascular diseases.

## Figures and Tables

**Table 1:** Key phenolic compound contents in the combined extract of black sticky rice and dill.

Detected Key Compounds	Detected Concentration (Milligrams per 1 g of Sample)
Gallic acid	0.027 ± 0.000
Rutin	1.166 ± 0.061
Quercetin	0.016 ± 0.001
Kaempferol	0.021 ± 0.000
Chlorogenic acid	3.984 ± 0.262
Caffeic acid	0.033 ± 0.001
Ferulic acid	0.017 ± 0.001
p-Coumaric acid	0.016 ± 0.000
Ellagic acid	2.028 ± 0.081
C3S	0.035 ± 0.002
C3G	0.165 ± 0.014
C3R	0.016 ± 0.000

C3S: *cyanidin* 3-O-sophoroside, C3G: *cyanidin* 3-O-glucoside, and C3R: *Cyanidin*-3-rutinoside.

**Table 2:** Compositions of the placebo capsule and the capsule containing the combined extract of dill and black sticky rice.

Ingredients	Formulation Code	
	(Dose of 600 mg)	Placebo
Dill and Black Sticky Rice Extract (mg)	600	-
Lactose Powder (mg)	99	99
Polyvinylpyrrolidone (PVP-K90) Powder (mg)	1	1
Tapioca Starch Powder	-	600
Total Weight (mg/capsule)	700	600

**Table 3:** Basic information of volunteers of all 3 groups before the commencement of product consumption (baseline) (mean ± SEM).

Parameters	Prior to the Consumption of the Product		
	Placebo	Combined Extract of Black Sticky Rice and Dill, 600 mg/day	Combined Extract of Black Sticky Rice and Dill, 1200 mg/day
Age (year) Gender (male/female)	63.15 ± 3.38 4/9	64.38 ± 1.69 (*p* = 0.521) 9/4	65.62 ± 2.12 (*p* = 0.368) 5/8
Body Temperature (°C)	36.58 ± 0.09	36.65 ± 0.09 (*p* = 0.856)	36.65 ± 0.07 (*p* = 0.587)
Heart Rate (beats/min)	80.77 ± 2.93	84.85 ± 4.30 (*p* = 0.411)	84.62 ± 3.80 (*p* = 0.537)
Respiratory Rate (breaths/min)	18.77 ± 0.28	19.23 ± 0.28 (*p* = 0.249)	18.92 ± 0.29 (*p* = 0.697)
Systolic BP (mmHg)	156.54 ± 5.66	153.77 ± 7.64 (*p* = 0.858)	157.08 ± 5.64 (*p* = 0.758)
Diastolic BP (mmHg)	83.77 ± 4.33	84.54 ± 4.03 (*p* = 0.980)	92.46 ± 2.69 (*p* = 0.150)
BMI (kg/m^2^)	24.07 ± 1.07	22.46 ± 0.92 (*p* = 0.174)	24.37 ± 0.89 (*p* = 0.959)

BMI = body mass index (kg/m^2^).

**Table 4:** Basic information on volunteers taking a placebo and the capsule containing the combined extract of black sticky rice and dill at the doses of 600 and 1200 mg/day at 1 week, 2 weeks, and 6 weeks of consumption compared with placebo (mean ± SEM).

Parameters	Placebo	Combined Extract of Black Sticky Rice and Dill, 600 mg/day	Combined Extract of Black Sticky Rice and Dill, 1200 mg/day
**1 week**			
Age (year) Gender (male/female)	63.15 ± 3.38 4/9	64.38 ± 1.69 (*p* = 0.521) 9/4	65.62 ± 2.12 (*p* = 0.368) 5/8
Body Temperature (°C)	36.36 ± 0.04	36.40 ± 0.06 (*p* = 0.515)	36.34 ± 0.05 (*p* = 0.874)
Heart Rate (beats/min)	76.77 ± 3.29	85.38 ± 4.79 (*p* = 0.166)	80.54 ± 4.20 (*p* = 0.208)
Respiratory Rate (breaths/min)	18.31 ± 0.38	18.77 ± 0.36 (*p* = 0.379)	18.77 ± 0.28 (*p* = 0.396)
Systolic BP (mmHg)	133.62 ± 3.55	142.23 ± 5.66 (*p* = 0.095)	140.15 ± 4.02 (*p* = 0.248)
Diastolic BP (mmHg)	77.38 ± 2.46	80.85 ± 3.77 (*p* = 0.488)	81.77 ± 2.51 (*p* = 0.328)
Body Weight (kg)	57.70 ± 3.10	61.11 ± 3.15 (*p* = 0.701)	60.86 ± 2.79 (*p* = 0.427)
BMI (kg/m^2^)	23.63 ± 1.19	22.53 ± 0.97 (*p* = 0.369)	24.36 ± 0.87 (*p* = 0.817)
**2 weeks**			
Age (year)	63.15 ± 3.38	64.31 ± 1.71 (*p* = 0.537)	65.62 ± 2.12 (*p* = 0.368)
Gender (Male/Female)	4/9	9/4	5/8
Body Temperature (°C)	36.40 ± 0.04	36.40 ± 0.06 (*p* = 0.946)	36.40 ± 0.07 (*p* = 0.340)
Heart Rate (beats/min)	84.00 ± 4.79	82.50 ± 5.29 (*p* = 0.644)	83.10 ± 1.88 (*p* = 0.384)
Respiratory Rate (breaths/min)	17.43 ± 0.53	18.50 ± 0.28 (*p* = 0.129)	18.80 ± 0.29 * (*p* = 0.015)
Systolic BP (mmHg)	133.29 ± 4.67	148.50 ± 12.50 (*p* = 0.843)	132.70 ± 4.76 (*p* = 0.514)
Diastolic BP (mmHg)	76.29 ± 2.72	88.50 ± 8.10 (*p* = 0.409)	78.00 ± 3.90 (*p* = 0.514)
Body Weight (kg)	53.46 ± 3.77	60.05 ± 2.13 (*p* = 0.235)	61.39 ± 3.20 (*p* = 0.292)
BMI (kg/m^2^)	21.41 ± 1.41	21.62 ± 1.00 (*p* = 1.000)	24.31 ± 1.01 (*p* = 0.420)
**6 weeks**			
Age (year)	63.15 ± 3.38	64.31 ± 1.71 (*p* = 0.537)	65.62 ± 2.12 (*p* = 0.368)
Gender (Male/Female)	4/9	9/4	5/8
Body Temperature (°C)	36.28 ± 0.09	36.65 ± 0.08 (*p* = 0.483)	36.32 ± 0.06 (*p* = 0.659)
Heart Rate (beats/min)	87.70 ± 3.80	72.50 ± 2.95 (*p* = 0.165)	78.80 ± 1.85 (*p* = 0.572)
Respiratory Rate (breaths/min)	19.60 ± 0.35	18.00 ± 0.45 (*p* = 0.157)	18.40 ±0.23 (*p* = 0.098)
Systolic BP (mmHg)	138.70 ± 3.84	144.50 ± 9.31 (*p* = 0.778)	134.30 ± 4.93 (*p* = 1.000)
Diastolic BP (mmHg)	77.80 ± 2.70	66.75 ± 1.18 (*p* = 0.644)	75.40 ± 2.28 (*p* = 0.589)
Body Weight (kg)	56.25 ± 3.57	60.78 ± 2.22 (*p* = 0.590)	61.77 ± 3.13 (*p* = 0.343)
BMI (kg/m^2^)	22.70 ± 1.36	22.49 ± 0.88 (*p* = 0.626)	24.47 ± 0.99 (*p* = 0.817)

BMI =body mass index (kg/m^2^).

**Table 5:** Illustrated clinical chemistry values of blood from volunteers across all three groups prior to product consumption (baseline) (mean + SEM), * *p* < 0.05; compared to placebo treated group.

Parameters	References	Placebo	Combined Extract of Black Sticky Rice and Dill, 600 mg/day	Combined Extract of Black Sticky Rice and Dill, 1200 mg/day
**Baseline**				
Hb	13.0–16.7 g/dL	12.62 ± 0.53	13.57 ± 0.36 (*p* = 0.191)	12.44 ± 0.33 (*p* = 0.538)
HCT	40.5–50.8%	38.03 ± 1.57	42.83 ± 0.60 (*p* = 0.305)	39.83 ± 0.81 (*p* = 0.473)
WBC	4.6–10.6 × 10^3^/μL	6.82 ± 0.69	8.52 ± 0.47 (*p* = 0.086)	7.77 ± 0.86 (*p* = 0.259)
Platelets	173–383 × 10^3^/μL	345.00 ± 80.85	271.25 ± 14.15 (*p* = 0.473)	276.90 ± 24.62 (*p* = 0.817)
RBC	4.7–6.2 × 10^6^/μL	4.39 ± 0.22	4.91 ± 0.07 * (*p* = 0.038)	4.47 ± 0.12 (*p* = 0.329)
MCV	80.0–97.8 fL	88.31 ± 3.79	87.23 ± 0.87 (*p* = 0.101)	89.60 ± 2.26 (*p* = 0.959)
MCH	25.2–32.0 pg	28.07 ± 1.26	28.85 ± 0.28 (*p* = 0.521)	28.58 ± 0.84 (*p* = 0.798)
MCHC	31.3–33.4 g/dL	31.74 ± 0.23	33.13 ± 0.10 (*p* = 0.329)	31.87 ± 0.31 (*p* = 0.939)
RDW	11.9–14.8%	14.63 ± 0.54	12.90 ± 0.20 (*p* = 0.662)	13.38 ± 0.19 (*p* = 0.555)
NE%	43.7–70.9%	62.40 ± 2.50	63.80 ± 2.60 (*p* = 0.878)	66.56 ± 3.29 (*p* = 0.293)
LY%	20.1–44.5%	25.92 ± 1.82	27.38 ± 2.74 (*p* = 0.457)	24.11 ± 2.66 (*p* = 0.330)
MO%	3.4–9.8%	6.17 ± 0.30	5.33 ± 0.41 * (*p* = 0.040)	5.52 ± 0.50 (*p* = 0.281)
EO%	0.7–9.2%	4.87 ± 1.77	2.98 ± 0.18 (*p* = 0.572)	3.31 ± 0.92 (*p* = 0.572)
BA%	0.0–2.6%	1.36 ± 0.65	0.53 ± 0.06 (*p* = 0.339)	0.50 ± 0.06 (*p* = 0.127)
**1 week**				
Hb	13.0–16.7 g/dL	12.62 ± 0.53	13.57 ± 0.36 (*p* = 0.191)	12.44 ± 0.33 (*p* = 0.538)
HCT	40.5–50.8%	38.03 ± 1.57	42.83 ± 0.60 (*p* = 0.305)	39.83 ± 0.81 (*p* = 0.473)
WBC	4.6–10.6 × 10^3^/μL	6.82 ± 0.69	8.52 ± 0.47 (*p* = 0.086)	7.77 ± 0.86 (*p* = 0.259)
Platelets	173–383 × 10^3^/μL	345.00 ± 80.85	271.25 ± 14.15 (*p* = 0.473)	276.90 ± 24.62 (*p* = 0.817)
RBC	4.7–6.2 × 10^6^/μL	4.39 ± 0.22	4.91 ± 0.07 * (*p* = 0.038)	4.47 ± 0.12 (*p* = 0.329)
MCV	80.0–97.8 fL	88.31 ± 3.79	87.23 ± 0.87 (*p* = 0.101)	89.60 ± 2.26 (*p* = 0.959)
MCH	25.2–32.0 pg	28.07 ± 1.26	28.85 ± 0.28 (*p* = 0.521)	28.58 ± 0.84 (*p* = 0.798)
MCHC	31.3–33.4 g/dL	31.74 ± 0.23	33.13 ± 0.10 (*p* = 0.329)	31.87 ± 0.31 (*p* = 0.939)
RDW	11.9–14.8%	14.63 ± 0.54	12.90 ± 0.20 (*p* = 0.662)	13.38 ± 0.19 (*p* = 0.555)
NE%	43.7–70.9%	62.40 ± 2.50	63.80 ± 2.60 (*p* = 0.878)	66.56 ± 3.29 (*p* = 0.293)
LY%	20.1–44.5%	25.92 ± 1.82	27.38 ± 2.74 (*p* = 0.457)	24.11 ± 2.66 (*p* = 0.330)
MO%	3.4–9.8%	6.17 ± 0.30	5.33 ± 0.41 * (*p* = 0.040)	5.52 ± 0.50 (*p* = 0.281)
EO%	0.7–9.2%	4.87 ± 1.77	2.98 ± 0.18 (*p* = 0.572)	3.31 ± 0.92 (*p* = 0.572)
BA%	0.0–2.6%	1.36 ± 0.65	0.53 ± 0.06 (*p* = 0.339)	0.50 ± 0.06 (*p* = 0.127)
**6 weeks**				
Hb	13.0–16.7 g/dL	12.38 ± 0.55	13.10 ± 0.37 (*p* = 0.301)	12.04 ± 0.34 (*p* = 0.479)
HCT	40.5–50.8%	37.74 ± 1.83	38.68 ± 1.17 (*p* = 0.430)	37.77 ± 1.02 (*p* = 0.496)
WBC	4.6–10.6 × 10^3^/μL	7.19 ± 0.82	7.73 ± 0.09 (*p* = 0.211)	6.13 ± 0.36 (*p* = 0.957)
Platelets	173–383 × 10^3^/μL	315.44 ± 66.30	231.25 ± 13.59 (*p* = 0.430)	239.40 ± 13.30 (*p* = 0.663)
RBC	4.7–6.2 × 10^6^/μL	4.39 ± 0.22	4.41 ± 0.10 (*p* = 0.103)	4.25 ± 0.10 (*p* = 0.480)
MCV	80.0–97.8 fL	86.94 ± 3.49	87.53 ± 0.90 (*p* = 0.115)	89.07 ± 2.27 (*p* = 0.786)
MCH	25.2–32.0 pg	27.71 ± 1.18	29.08 ± 0.32 (*p* = 0.341)	28.79 ± 0.85 (*p* = 0.828)
MCHC	31.3–33.4 g/dL	28.53 ± 2.67	33.20 ± 0.28 (*p* = 0.253)	32.30 ± 0.39 (*p* = 0.723)
RDW	11.9–14.8%	15.00 ± 0.70	13.23 ± 0.23 (*p* = 0.892)	13.34 ± 0.26 (*p* = 0.624)
NE%	43.7–70.9%	64.04 ± 3.70	62.83 ± 1.74 (*p* = 0.683)	58.03 ± 2.48 (*p* = 0.463)
LY%	20.1–44.5%	24.74 ± 2.51	26.83 ± 1.92 (*p* = 0.978)	29.54 ± 1.64 (*p* = 0.624)
MO%	3.4–9.8%	5.84 ± 0.72	6.33 ± 0.76 (*p* = 0.231)	5.55 ± 0.29 (*p* = 0.201)
EO%	0.7–9.2%	4.71 ± 1.59	3.30 ± 0.30 (*p* = 0.744)	6.24 ± 1.18 (*p* = 0.369)
BA%	0.0–2.6%	0.66 ± 0.10	0.48 ± 0.03 (*p* = 0.154)	0.64 ± 0.08 (*p* = 0.528)

Hemoglobin (Hb), hematocrit (HCT), white blood cell (WBC), platelet (PLT), red blood cell (RBC), mean corpuscular volume (MCV), mean corpuscular hemoglobin (MCH), red cell distribution width (RDW), mean corpuscular hemoglobin concentration (MCHC), neutrophil (NE), lymphocytes (LY), monocytes (MO), eosinophils (EO), and basophils (BA).

**Table 6:** Illustrated clinical chemistry values of blood from volunteers across all three groups prior to product consumption (baseline) (mean ± SEM).

Parameters	References	Placebo	Combined Extract of Black Sticky Rice and Dill, 600 mg/day	Combined Extract of Black Sticky Rice and Dill, 1200 mg/day
Blood Glucose	<180 mg/dL	111.85 ± 8.16	128.00 ± 12.78 (*p* = 0.412)	99.62 ± 5.19 (*p* = 0.383)
BUN	5.8–19.1 mg/dL	11.8 ± 1.15	11.94 ± 1.52 (*p* = 0.898)	12.42 ± 1.72 (*p* = 0.918)
Creatinine	0.5–1.5 mEq/L	0.83 ± 0.06	1.01 ± 0.10 (*p* = 0.199)	1.08 ± 0.13 (*p* = 0.166)
Uric Acid	2.7–7.0 mg/dL	5.17 ± 0.47	5.85 ± 0.47(*p* = 0.446)	5.60 ± 0.33 (*p* = 0.681)
Sodium	130–147 mEq/L	140 ± 0.71	139.69 ± 0.47 (*p* = 0.480)	140.69 ± 0.62 (*p* = 0.480)
Potassium	3.4–4.7 mEq/L	4.28 ± 0.19	3.82 ± 0.26 (*p* = 0.471)	4.02 ± 0.10 (*p* = 0.207)
Bicarbonate	20.6–28.3 mEq/L	21.65 ± 0.55	22.06 ± 0.62 (*p* = 0.797)	21.40 ± 0.46 (*p* = 0.959)
Chloride	96–107 mEq/L	102.23 ± 0.94	101.77 ± 0.80 (*p* = 0.606)	102.54 ± 1.05 (*p* = 0.836)
Total Protein	6.5–8.8 g/dL	6.89 ± 0.19	6.69 ± 0.17 (*p* = 0.396)	6.75 ± 0.14 (*p* = 0.571)
Albumin	3.8–5.4 g/dL	3.88 ± 0.10	3.92 ± 0.10 (*p* = 0.959)	3.98 ± 0.05 (*p* = 0.623)
Globulin	2.6–3.4 g/dL	3.03 ± 0.21	2.78 ± 0.12 (*p* = 0.471)	2.77 ± 0.13 (*p* = 0.440)
Total Bilirubin	0.3–1.5 mg/dL	0.63 ± 0.08	0.52 ± 0.05 (*p* = 0.375)	0.75 ± 0.11 (*p* = 0.465)
Direct Bilirubin	0.0–0.5 mg/dL	0.25 ± 0.05	0.22 ± 0.02 (*p* = 0.931)	0.28 ± 0.04 (*p* = 0.290)
ALT	4–36 U/L	28.62 ± 10.23	19.62 ± 3.43(*p* = 0.758)	23.92 ± 3.48 (*p* = 0.521)
AST	12–32 U/L	52.46 ± 26.01	21.85 ± 1.68 (*p* = 0.877)	26.46 ± 2.98 (*p* = 0.537)
ALP	42–121 U/L	85.54 ± 13.52	86.46 ± 7.49 (*p* = 0.248)	78.08 ± 6.59 (*p* = 0.797)
Amylase	25–125 U/L	69.38 ± 8.31	79.75 ± 9.19 (*p* = 0.414)	78.69 ± 9.80 (*p* = 0.538)
LDH	0–250 U/L	200.08 ± 29.54	167.25 ± 10.60 (*p* = 0.414)	214.38 ± 26.03 (*p* = 0.457)
CK	38–170 U/L	72.00 ± 9.14	105.92 ± 21.39 (*p* = 0.242)	95.77 ± 13.13 (*p* = 0.158)
Gamma-GT	0–50 U/L	78.31 ± 32.79	44.75 ± 6.15 (*p* = 0.220)	63.77 ± 23.56 (*p* = 0.939)
Thyroxine (T4)	4.5–11.7 μg/dL	7.56 ± 0.66	6.93 ± 0.22 (*p* = 0.568)	7.50 ± 0.31 (*p* = 0.980)
Triiodothyronine (T3)	80–180 ng/dL	88.15 ± 6.46	91.44 ± 3.92 (*p* = 0.480)	90.38 ± 5.25 (*p* = 0.778)
TSH	0.2–4.2 mlU/L	1.12 ± 0.11	1.30 ± 0.18 (*p* = 0.550)	1.02 ± 0.2 (*p* = 0.259)
Estradiol	pg/mL	20.75 ± 2.70	23.94 ± 4.93 (*p* = 1.000)	17.93 ± 1.92 (*p* = 0.637)
Testosterone	1.93–8.36 ng/mL	2.24 ± 0.30	4.98 ± 0.48 (*p* = 0.053)	4.48 ± 0.6 (*p* = 0.121)
Cortisol In Blood	6.2–19.4 μg/dL	7.45 ± 1.15	7.80 ± 0.26 (*p* = 0.505)	8.62 ± 0.57 (*p* = 0.831)
LH	7.7–58.5 mIU/mL	34.08 ± 6.25	18.89 ± 4.92 (*p* = 0.092)	25.30 ± 4.99 (*p* = 0.317)
FSH	25.8–134.8 mlU/mL	71.38 ± 9.75	43.18 ± 9.08 (*p* = 0.129)	68.53 ± 6.50 (*p* = 0.657)
Cholesterol	127–262 mg/dL	165.77 ± 14.57	205.15 ± 16.88 (*p* = 0.174)	161.92 ± 8.39 (*p* = 0.878)
Triglyceride	10–200 mg/dL	112.69 ± 15.51	155.23 ± 21.53 (*p* = 0.073)	109.69 ± 11.94 (*p* = 0.700)
HDL-C	>35 mg/dL	39.31 ± 3.86	40.00 ± 1.93 (*p* = 0.959)	47.31 ± 3.63 (*p* = 0.199)
Atherogenic Index (AIP)	<0.11	0.47 ± 0.09	0.55 ± 0.07 (*p* = 0.228)	0.34 ± 0.08 (*p* = 0.442)
LDL-Chol (DIRECT)	10–150 mg/dL	110.92 ± 12.67	123.85 ± 13.31 (*p* = 0.505)	118.00 ± 10.19 (*p* = 0.644)

BUN = blood urea nitrogen, ALT = alanine aminotransferase, AST = aspartate aminotransferase, LDH = lactic dehydrogenase, CK = creatine kinase, TSH = thyroid-stimulating hormone, LH = luteinizing hormone, FSH = follicle-stimulating hormone, HDL-C = high-density lipoprotein cholesterol; LDL-CHOL = low-density lipoprotein cholesterol, AI = atherogenic index.

**Table 7:** Illustrated clinical chemistry values of blood from volunteers across all three groups prior to product consumption (baseline) (mean + SEM), * *p* < 0.05; compared to placebo treated group.

Parameters	References	Placebo	Combined Extract of Black Sticky Rice and Dill, 600 mg/day	Combined Extract of Black Sticky Rice and Dill, 1200 mg/day
**1 week**				
Blood Glucose	<180 mg/dL	122.92 ± 14.74	168.62 ± 26.69 (*p* = 0.143)	132.08 ± 9.21 (*p* = 0.228)
BUN	5.8–19.1 mg/dL	19.96 ± 7.55	13.99 ± 1.72 (*p* = 0.858)	14.07 ± 1.46 (*p* = 0.817)
Creatinine	0.5–1.5 mEq/L	0.80 ± 0.07	1.01 ± 0.09 (*p* = 0.065)	1.15 ± 0.15 * (*p* = 0.021)
Uric Acid	2.7–7.0 mg/dL	5.12 ± 0.36	6.28 ± 0.54 (*p* = 0.182)	5.85 ± 0.28 (*p* = 0.182)
Sodium	130–147 mEq/L	140.31 ± 0.69	140.92 ± 0.47 (*p* = 0.549)	141.08 ± 0.40 (*p* = 0.463)
Potassium	3.4–4.7 mEq/L	4.13 ± 0.14	4.11 ± 0.10 (*p* = 1.000)	4.23 ± 0.09 (*p* = 0.699)
Bicarbonate	20.6–28.3 mEq/L	24.08 ± 0.80	24.78 ± 0.58 (*p* = 0.488)	23.98 ± 0.51 (*p* = 0.898)
Chloride	96–107 mEq/L	99.54 ± 1.11	93.00 ± 6.96 (*p* = 0.680)	100.85 ± 0.77 (*p* = 0.378)
Total Protein	6.5–8.8 g/dL	7.18 ± 0.15	7.07 ± 0.13 (*p* = 0.589)	7.32 ± 0.12 (*p* = 0.354)
Albumin	3.8–5.4 g/dL	4.1 ± 0.14	4.17 ± 0.12 (*p* = 0.777)	4.38 ± 0.08 (*p* = 0.206)
Globulin	2.6–3.4 g/dL	3.08 ± 0.20	2.90 ± 0.10 (*p* = 0.797)	2.94 ± 0.16 (*p* = 0.857)
Total Bilirubin	0.3–1.5 mg/dL	0.48 ± 0.07	0.38 ± 0.04 (*p* = 0.416)	0.50 ± 0.06 (*p* = 0.677)
Direct Bilirubin	0.0–0.5 mg/dL	0.26 ± 0.05	0.30 ± 0.14 (*p* = 0.157)	0.23 ± 0.02 (*p* = 0.678)
ALT	4–36 U/L	28.31 ± 9.09	27.23 ± 5.35 (*p* = 0.625)	27.31 ± 3.36 (*p* = 0.227)
AST	12–32 U/L	48.54 ± 23.05	24.69 ± 2.61 (*p* = 0.939)	23.85 ± 1.74 (*p* = 0.797)
ALP	42–121 U/L	91.15 ± 13.76	95.15 ± 5.67 (*p* = 0.077)	95.69 ± 10.94 (*p* = 0.700)
Amylase	25–125 U/L	78.69 ± 8.26	85.23 ± 10.40 (*p* = 0.817)	96.00 ± 10.68 (*p* = 0.191)
LDH	0–250 U/L	210.38 ± 19.91	179.54 ± 6.97 (*p* = 0.248)	206.31 ± 14.64 (*p* = 0.858)
CK	38–170 U/L	85.85 ± 10.90	104.66 ± 17.49 (*p* = 0.473)	118.00 ± 31.71 (*p* = 0.342)
Gamma-GT	0–50 U/L	68.69 ± 25.23	49.92 ± 8.36 (*p* = 0.383)	72.54 ± 22.19 (*p* = 0.473)
Thyroxine (T4)	4.5–11.7 μg/dL	8.71 ± 0.66	7.90 ± 0.34 (*p* = 0.144)	8.30 ± 0.37 (*p* = 0.521)
Triiodothyronine (T3)	80–180 ng/dL	105.32 ± 6.97	100.97 ± 5.17 (*p* = 0.739)	97.84 ± 4.25 (*p* = 0.249)
TSH	0.2–4.2 mlU/L	1.38 ± 0.19	1.50 ± 0.18 (*p* = 0.532)	1.04 ± 0.21 (*p* = 0.065)
Estradiol	pg/mL	15.45 ± 1.05	19.25 ± 4.97 (*p* = 0.398)	18.97 ± 1.75 (*p* = 0.242)
Testosterone	1.93–8.36 ng/mL	4.13 ± 0.37	4.41 ± 0.74 (*p* = 0.732)	15.27 ± 7.41 (*p* = 0.606)
LH	7.7–58.5 mIU/mL	37.13 ± 7.01	18.86 ± 4.41 (*p* = 0.057)	26.79 ± 5.32 (*p* = 0.273)
FSH	25.8–134.8 mlU/mL	81.31 ± 10.86	66.25 ± 3.67 (*p* = 0.396)	76.17 ± 7.46 (*p* = 0.845)
Cholesterol	127–262 mg/dL	132.92 ± 8.84	157.92 ± 12.25 (*p* = 0.158)	138.15 ± 6.50 (*p* = 0.538)
Triglyceride	10–200 mg/dL	112.85 ± 13.82	135.38 ± 14.48 (*p* = 0.281)	124.23 ± 16.74 (*p* = 0.644)
HDL-C	>35 mg/dL	42.15 ± 2.66	44.08 ± 2.37 (*p* = 0.554)	49.15 ± 4.26 (*p* = 0.248)
Atherogenic Index (AIP)	<0.11	0.40 ± 0.07	0.46 ± 0.06 (*p* = 0.472)	0.38 ± 0.07 (*p* = 0.608)
LDL-Chol (DIRECT)	10–150 mg/dL	73.23 ± 7.91	102.85 ± 9.44 * (*p* = 0.031)	72.00 ± 4.35 (*p* = 0.898)
**6 weeks**				
Blood Glucose	<180 mg/dL	135.08 ± 15.24	165.77 ± 28.56 (*p* = 0.383)	127.54 ± 9.18 (*p* = 0.739)
BUN	5.8–19.1 mg/dL	11.58 ± 0.68	12.86 ± 1.22 (*p* = 1.000)	14.26 ± 1.58 (*p* = 0.412)
Creatinine	0.5–1.5 mEq/L	0.87 ± 0.07	1.08 ± 0.11(*p* = 0.130)	1.19 ± 0.17 (*p* = 0.144)
Uric Acid	2.7–7.0 mg/dL	4.98 ± 0.32	6.02 ± 0.54 (*p* = 0.259)	5.78 ± 0.34 (*p* = 0.368)
Sodium	130–147 mEq/L	140.31 ± 0.69	140.23 ± 0.80 (*p* = 0.917)	140.92 ± 0.49 (*p* = 0.533)
Potassium	3.4–4.7 mEq/L	4.12 ± 0.12	4.04 ± 0.12 (*p* = 0.485)	4.14 ± 0.12 (*p* = 0.938)
Bicarbonate	20.6–28.3 mEq/L	24.08 ± 1.03	24.52 ± 0.33 (*p* = 0.555)	24.52 ± 0.59 (*p* = 0.397)
Chloride	96–107 mEq/L	100.00 ± 0.95	100.38 ± 0.81 (*p* = 0.877)	100.08 ± 0.84 (*p* = 0.737)
Total Protein	6.5–8.8 g/dL	7.34 ± 0.16	7.12 ± 0.12 (*p* = 0.409)	7.15 ± 0.08 (*p* = 0.502)
Albumin	3.8–5.4 g/dL	4.23 ± 0.13	4.20 ± 0.09 (*p* = 0.738)	4.32 ± 0.09 (*p* = 0.757)
Globulin	2.6–3.4 g/dL	3.10 ± 0.21	2.93 ± 0.11 (*p* = 0.643)	2.83 ± 0.12 (*p* = 0.409)
Total Bilirubin	0.3–1.5 mg/dL	0.52 ± 0.07	0.45 ± 0.05 (*p* = 0.544)	0.62 ± 0.10 (*p* = 0.448)
Direct Bilirubin	0.0–0.5 mg/dL	0.25 ± 0.04	0.19 ± 0.02 (*p* = 0.260)	0.27 ± 0.04 (*p* = 0.646)
ALT	4–36 U/L	31.62 ± 8.42	21.31 ± 2.76 (*p* = 0.797)	31.00 ± 5.84 (*p* = 0.817)
AST	12–32 U/L	49.08 ± 19.59	21.77 ± 1.69 (*p* = 0.355)	24.85 ± 2.62 (*p* = 0.739)
ALP	42–121 U/L	99.38 ± 16.2	96.23 ± 8.76 (*p* = 0.555)	127.62 ± 38.97 (*p* = 0.644)
Amylase	25–125 U/L	77.08 ± 8.92	89.00 ± 9.66 (*p* = 0.158)	97.46 ± 12.65 (*p* = 0.259)
LDH	0–250 U/L	205.77 ± 17.70	170.08 ± 9.52 (*p* = 0.077)	188.54 ± 11.05 (*p* = 0.538)
CK	38–170 U/L	112.77 ± 38.63	121.15 ± 29.36 (*p* = 0.797)	106.54 ± 21.86 (*p* = 0.719)
Gamma-GT	0–50 U/L	87.38 ± 32.13	48.92 ± 7.28 (*p* = 0.355)	88.77 ± 45.42 (*p* = 0.898)
Thyroxine (T4)	4.5–11.7 μg/dL	7.87 ± 0.66	7.32 ± 0.35 (*p* = 0.663)	7.58 ± 0.39 (*p* = 0.939)
Triiodothyronine (T3)	80–180 ng/dL	106.87 ± 5.96	104.10 ± 5.74 (*p* = 0.573)	110.22 ± 5.67 (*p* = 0.739)
TSH	0.2–4.2 mlU/L	1.30 ± 0.18	1.38 ± 0.23 (*p* = 0.858)	1.07 ± 0.10 (*p* = 0.521)
Estradiol	pg/mL	15.86 ± 1.66	22.42 ± 0.00 (*p* = 0.245)	14.04 ± 1.49 (*p* = 0.643)
Testosterone	1.93–8.36 ng/mL	5.12 ± 0.51	4.39 ± 0.48 (*p* = 0.644)	5.27 ± 0.75 (*p* = 0.655)
LH	7.7–58.5 mIU/mL	39.22 ± 6.99	18.02 ± 4.21 * (*p* = 0.026)	29.06 ± 5.77 (*p* = 0.293)
FSH	25.8–134.8 mlU/mL	84.33 ± 12.21	67.40 ± 2.77 (*p* = 0.203)	76.78 ± 7.15 (*p* = 0.450)
Cholesterol	127–262 mg/dL	131.92 ± 12.28	151.62 ± 11.92 (*p* = 0.356)	146.00 ± 6.95 (*p* = 0.383)
Triglyceride	10–200 mg/dL	122.46 ± 21.36	130.08 ± 16.44 (*p* = 0.590)	134.15 ± 12.02 (*p* = 0.305)
HDL-C	>35 mg/dL	41.92 ± 2.04	46.15 ± 2.05 (*p* = 0.236)	46.38 ± 3.20 (*p* = 0.504)
Atherogenic Index (AIP)	<0.11	0.41 ± 0.07	0.41 ± 0.07 (*p* = 0.778)	0.45 ± 0.05 (*p* = 0.457)
LDL-Chol (DIRECT)	10–150 mg/dL	72.46 ± 9.96	86.77 ± 9.56 (*p* = 0.383)	80.92 ± 5.54 (*p* = 0.317)

**Table 8:** Effect of the combined extract of black sticky rice and dill on the control of movement, as assessed using the National Institute of Health Stroke Score (NIHSS). Data are presented as mean ± standard deviation (SD). * *p*-value < 0.05, compared to the placebo control group.

Duration after Consumption of the Product	Experimental Group	Percentage Change in Total NIHSS
1 week	Placebo	−36.03 + 13.96
	The combined extract of black sticky rice and dill, 600 mg/day	−50.15 + 12.16 (*p* = 0.433)
	The combined extract of black sticky rice and dill, 1200 mg/day	−87.50 + 12.50 (*p* = 0.045) *
2 weeks	Placebo	−62.55 + 14.24
	The combined extract of black sticky rice and dill, 600 mg/day	−58.03 + 13.00 (*p* = 0.808)
	The combined extract of black sticky rice and dill, 1200 mg/day	−87.50 + 12.50 (*p* = 0.332)
6 weeks	Placebo	−73.48 + 13.71
	The combined extract of black sticky rice and dill, 600 mg/day	−90.15 + 5.36 (*p* = 0.241)
	The combined extract of black sticky rice and dill, 1200 mg/day	−93.75 + 6.25 (*p* = 0.297)

**Table 9:** Results of the daily routine assessment (Barthel Index) of volunteers prior to the consumption of the product (baseline) (mean + SEM). * *p* < 0.05 when compared to the placebo.

Parameters	Placebo	Combined Extract of Black Sticky Rice and Dill, 600 mg/day	Combined Extract of Black Sticky Rice and Dill, 1200 mg/day
**Prior to the consumption of the product**			
1. Feeding	9.23 ± 0.52	10.00 ± 0.00 (*p* = 0.149)	10.00 ± 0.00 (*p* = 0.149)
2. Transfer	12.69 ± 0.92	13.85 ± 0.61 (*p* = 0.356)	13.85 ± 0.83 (*p* = 0.235)
3. Walking	13.46 ± 0.67	13.85 ± 0.61 (*p* = 0.665)	13.85 ± 0.61 (*p* = 0.665)
4. Dressing	8.08 ± 0.70	9.62 ± 0.38 (*p* = 0.068)	10.00 ± 0.00 * (*p* = 0.015)
5. Bathing	3.85 ± 0.61	4.62 ± 0.38 (*p* = 0.286)	5.00 ± 0.00 (*p* = 0.071)
6. Grooming	4.23 ± 0.52	5.00 ± 0.00 (*p* = 0.149)	5.00 ± 0.00 (*p* = 0.149)
7. Toilet use	7.31 ± 1.22	8.85 ± 0.61 (*p* = 0.468)	9.62 ± 0.38 (*p* = 0.117)
8. Bowel control	9.62 ± 0.38	9.62 ± 0.38 (*p* = 1.000)	10.00 ± 0.00 (*p* = 0.317)
9. Bladder control	8.46 ± 0.87	8.85 ± 0.83 (*p* = 0.654)	9.62 ± 0.38 (*p* = 0.270)
10. Stairs	7.31 ± 0.92	7.31 ± 1.08 (*p* = 0.862)	8.46 ± 0.87 (*p* = 0.270)
**1 week**			
1. Feeding	10.00 ± 0.00	9.62 ± 0.38 (*p* = 0.317)	10.00 ± 0.00 (*p* = 1.000)
2. Transfer	13.46 ± 0.87	13.46 ± 0.67 (*p* = 0.765)	14.62 ± 0.38 (*p* = 0.270)
3. Walking	13.46 ± 0.87	14.23 ± 0.52 (*p* = 0.575)	14.62 ± 0.38 (*p* = 0.270)
4. Dressing	8.46 ± 0.87	9.23 ± 0.52 (*p* = 0.575)	10.00 ± 0.00 (*p* = 0.071)
5. Bathing	4.62 ± 0.38	4.23 ± 0.52 (*p* = 0.547)	5.00 ± 0.00 (*p* = 0.317)
6. Grooming	5.00 ± 0.00	4.62 ± 0.38 (*p* = 0.317)	5.00 ± 0.00 (*p* = 1.000)
7. Toilet use	8.85 ± 0.83	8.85 ± 0.83 (*p* = 1.000)	10.00 ± 0.00 (*p* = 0.149)
8. Bowel control	10.00 ± 0.00	9.62 ± 0.38 (*p* = 0.317)	10.00 ± 0.00 (*p* = 1.000)
9. Bladder control	9.62 ± 0.38	9.23 ± 0.52 (*p* = 0.547)	10.00 ± 0.00 (*p* = 0.317)
10. Stairs	7.69 ± 0.92	8.08 ± 1.06 (*p* = 0.549)	9.23 ± 0.52 (*p* = 0.174)
**2 weeks**			
1. Feeding	10.00 ± 0.00	10.00 ± 0.00(*p* = 1.000)	10.00 ± 0.00 (*p* = 1.000)
2. Transfer	13.85 ± 0.83	14.23 ± 0.52 (*p* = 0.935)	15.00 ± 0.00 (*p* = 0.149)
3. Walking	14.23 ± 0.77	14.23 ± 0.52 (*p* = 0.611)	15.00 ± 0.00 (*p* = 0.317)
4. Dressing	9.23 ± 0.77	9.23 ± 0.52 (*p* = 0.611)	10.00 ± 0.00 (*p* = 0.317)
5. Bathing	4.62 ± 0.38	4.23 ± 0.52 (*p* = 0.547)	5.00 ± 0.00 (*p* = 0.317)
6. Grooming	5.00 ± 0.00	5.00 ± 0.00 (*p* = 1.000)	5.00 ± 0.00 (*p* = 1.000)
7. Toilet use	8.46 ± 0.87	9.23 ± 0.52 (*p* = 0.575)	10.00 ± 0.00 (*p* = 0.071)
8. Bowel control	9.23 ± 0.77	9.62 ± 0.38 (*p* = 0.956)	10.00 ± 0.00 (*p* = 0.317)
9. Bladder control	9.62 ± 0.38	9.23 ± 0.52(*p* = 0.547)	10.00 ± 0.00 (*p* = 0.317)
10. Stairs	7.69 ± 0.92	8.08 ± 1.06 (*p* = 0.549)	9.62 ± 0.38 (*p* = 0.064)
**6 weeks**			
1. Feeding	10.00 ± 0.00	10.00 ± 0.00 (*p* = 1.000)	10.00 ± 0.00 (*p* = 1.000)
2. Transfer	14.23 ± 0.52	14.23 ± 0.52 (*p* = 1.000)	15.00 ± 0.00 (*p* = 0.149)
3. Walking	14.23 ± 0.77	14.23 ± 0.52 (*p* = 0.611)	15.00 ± 0.00 (*p* = 0.317)
4. Dressing	10.00 ± 0.00	9.23 ± 0.52 (*p* = 0.149)	10.00 ± 0.00 (*p* = 1.000)
5. Bathing	5.00 ± 0.00	4.62 ± 0.38 (*p* = 0.317)	5.00 ± 0.00 (*p* = 1.000)
6. Grooming	5.00 ± 0.00	5.00 ± 0.00 (*p* = 1.000)	5.00 ± 0.00 (*p* = 1.000)
7. Toilet use	9.23 ± 0.52	9.23 ± 0.52 (*p* = 1.000)	10.00 ± 0.00 (*p* = 0.149)
8. Bowel control	10.00 ± 0.00	10.00 ± 0.00 (*p* = 1.000)	10.00 ± 0.00 (*p* = 1.000)
9. Bladder control	9.62 ± 0.38	9.62 ± 0.38 (*p* = 1.000)	10.00 ± 0.00 (*p* = 0.317)
10. Stairs	8.46 ± 0.87	8.85 ± 0.83 (*p* = 0.654)	10.00 ± 0.00 (*p* = 0.071)

**Table 10:** Results of the disability level assessment in volunteers post-stroke, prior to the consumption of the product (baseline) (mean + SEM), * *p* < 0.05; compared to placebo treated group.

Parameters	Placebo	600 mg Capsules of Black Sticky Rice Mixed with Dill Extract	1200 mg Capsules of Black Sticky Rice Mixed with Dill Extract
**Prior to the consumption of the product**			
0: No symptoms at all	0.00 ± 0.00	0.00 ± 0.00 (*p =* 1.000)	0.00 ± 0.00 (*p =* 1.000)
1: No significant disability	0.38 ± 0.14	0.62 ± 0.14 (*p =* 0.249)	0.23 ± 0.12 (*p =* 0.405)
2: Slight disability	0.46 ± 0.24	0.00 ± 0.00 (*p =* 0.071)	0.15 ± 0.15 (*p =* 0.286)
3: Moderate disability	0.46 ± 0.31	0.23 ± 0.23 (*p =* 0.547)	0.23 ± 0.23 (*p =* 0.547)
4: Moderately severe disability	0.62 ± 0.42	0.62 ± 0.42 (*p =* 1.000)	0.31 ± 0.31 (*p =* 0.547)
5: Severe disability	0.00 ± 0.00	0.00 ± 0.00 (*p =* 1.000)	0.00 ± 0.00 (*p =* 1.000)
6: Dead	0.00 ± 0.00	0.00 ± 0.00 (*p =* 1.000)	0.00 ± 0.00 (*p =* 1.000)
**1 week**			
0: No symptoms at all	0.00 ± 0.00	0.00 ± 0.00 (*p =* 1.000)	0.00 ± 0.00 (*p =* 1.000)
1: No significant disability	0.31 ± 0.13	0.46 ± 0.14 (*p =* 0.429)	0.00 ± 0.00 * (*p =* 0.033)
2: Slight disability	0.46 ± 0.33	0.31 ± 0.21 (*p =* 0.935)	0.00 ± 0.00 (*p =* 0.149)
3: Moderate disability	0.23 ± 0.23	0.00 ± 0.00 (*p =* 0.317)	0.46 ± 0.31 (*p =* 0.547)
4: Moderately severe disability	0.31 ± 0.31	0.31 ± 0.31 (*p =* 1.000)	0.00 ± 0.00 (*p =* 0.317)
5: Severe disability	0.00 ± 0.00	0.00 ± 0.00 (*p =* 1.000)	0.00 ± 0.00 (*p =* 1.000)
6: Dead	0.00 ± 0.00	0.00 ± 0.00 (*p =* 1.000)	0.00 ± 0.00 (*p =* 1.000)
**2 weeks**			
0: No symptoms at all	0.08 ± 0.08	0.00 ± 0.00 (*p =* 0.317)	0.00 ± 0.00 (*p =* 0.317)
1: No significant disability	0.15 ± 0.10	0.31 ± 0.13 (*p =* 0.361)	0.00 ± 0.00 (*p =* 0.149)
2: Slight disability	0.00 ± 0.00	0.15 ± 0.15 (*p =* 0.317)	0.15 ± 0.15 (*p =* 0.317)
3: Moderate disability	0.23 ± 0.23	0.00 ± 0.00 (*p =* 0.317)	0.23 ± 0.23 (*p =* 1.000)
4: Moderately severe disability	0.31 ± 0.31	0.31 ± 0.31 (*p =* 1.000)	0.00 ± 0.00 (*p =* 0.317)
5: Severe disability	0.00 ± 0.00	0.00 ± 0.00 (*p =* 1.000)	0.00 ± 0.00 (*p =* 1.000)
6: Dead	0.00 ± 0.00	0.00 ± 0.00 (*p =* 1.000)	0.00 ± 0.00 (*p =* 1.000)
**6 weeks**			
0: No symptoms at all	0.00 ± 0.00	0.00 ± 0.00 (*p =* 1.000)	0.00 ± 0.00 (*p =* 1.000)
1: No significant disability	0.15 ± 0.10	0.15 ± 0.10 (*p =* 1.000)	0.15 ± 0.10 (*p =* 1.000)
2: Slight disability	0.15 ± 0.15	0.00 ± 0.00 (*p =* 0.317)	0.00 ± 0.00 (*p =* 0.317)
3: Moderate disability	0.00 ± 0.00	0.00 ± 0.00 (*p =* 1.000)	0.00 ± 0.00 (*p =* 1.000)
4: Moderately severe disability	0.31 ± 0.31	0.31 ± 0.31 (*p =* 1.000)	0.00 ± 0.00 (*p =* 0.317)
5: Severe disability	0.00 ± 0.00	0.00 ± 0.00 (*p =* 1.000)	0.00 ± 0.00 (*p =* 1.000)
6: Dead	0.00 ± 0.00	0.00 ± 0.00 (*p =* 1.000)	0.00 ± 0.00 (*p =* 1.000)

**Table 11:** Effect of the combined extract of black sticky rice and dill on ischemic stroke markers, including VCAM-1, MMP-9, vWF, and S100β, * *p* < 0.05, ** *p* < 0.01; compared to placebo treated group.

Parameters	Placebo	Combined Extract of Black Sticky Rice and Dill, 600 mg/day	Combined Extract of Black Sticky Rice and Dill, 1200 mg/day
**Prior to consumption**			
VCAM1 (ng/mL)	192.10 ± 6.77	189.53 ± 7.61 (*p* = 0.778)	190.04 ± 7.44 (*p* = 0.939)
MMP-9 (pg/mL)	116.06 ± 9.52	112.05 ± 8.86 (*p* = 0.701)	102.82 ± 8.94 (*p* = 0.270)
vWF (mlU/mL)	77.37 ± 0.90	78.46 ± 1.61 (*p* = 0.191)	77.48 ± 1.21 (*p* = 0.980)
S100β (pg/mL)	86.83 ± 4.66	84.33 ± 6.18 (*p* = 0.778)	102.02 ± 13.69 (*p* = 0.626)
**After consumption of the product for 1 week**			
VCAM1 (ng/mL)	217.43 ± 6.07	223.37 ± 6.34 (*p* = 0.477)	199.07 ± 4.98 * (*p* = 0.036)
MMP-9 (pg/mL)	129.82 ± 5.61	113.66 ± 5.93 (*p* = 0.059)	105.63 ± 5.99 ** (*p* = 0.006)
vWF (mlU/mL)	92.23 ± 0.98	91.88 ± 1.04 (*p* = 0.798)	90.38 ± 0.63 (*p* = 0.174)
S100β (pg/mL)	94.60 ± 6.06	94.02 ± 4.47 (*p* = 0.644)	95.27 ± 3.35 (*p* = 0.898)
**After consumption of the product for 6 weeks**			
VCAM1 (ng/mL)	256.78 ± 6.77	222.20 ± 7.71 ** (*p* = 0.003)	230.85 ± 8.65 * (*p* = 0.024)
MMP-9 (pg/mL)	110.032 ± 6.02	125.43 ± 5.56 (*p* = 0.07)	115.39 ± 5.88 (*p* = 0.520)
vWF (mlU/mL)	95.59 ± 0.57	95.41 ± 0.56 (*p* = 0.817)	94.79 ± 0.43 (*p* = 0.317)
S100β (pg/mL)	109.51 ± 5.60	103.94 ± 4.09 (*p* = 0.379)	95.24 ± 3.19 * (*p* = 0.032)

## Data Availability

Data will be provided upon request due to the petty patent registration.

## Supplementary Materials

The following supporting information can be downloaded at https://www.mdpi.com/article/10.3390/nu16223946/s1, Table S1. The combination index (CI) of the combined extract is provided in the Supplementary Materials.
