# Supplementary material for: A Combined Extract Derived from Black Sticky Rice and Dill Improves Clinical Symptoms and Ischemic Stroke Biomarkers in Transient Ischemic Attack and Ischemic Stroke Patients
Source: Nutrients. 2024 Nov 19;16(22):3946. doi: 10.3390/nu16223946 (PMC11597798; doi:10.3390/nu16223946)
Supplement: Supplementary file 1 [file nutrients-16-03946-s001.zip › nutrients-3288367-supplementary.pdf]

Table S1 Combination index of the combined extract of black sticky rice and dill

Parameters	Units	Black sticky rice	Dill	Black sticky rice and Dill.	CI
DPPH	EC_{50} (mg/ml)	0.123 ± 0.01	0.065 ± 0.02	0.014±0.003 [aa]	0.319
FRAP	EC_{50} (mg/ml)	2.56 ± 0.37	2.99 ± 0.055	1.19 ± 0.24 [a,b]	0.863
COX-2 inhibition	EC_{50} (mg/ml)	67.85± 2.05	9.37± 1.18	1.73 ± 0.44[aaa]	0.051
